# Evidence for divergence of DNA methylation maintenance and a conserved inhibitory mechanism from DNA demethylation in chickens and mammals

**DOI:** 10.1007/s13258-021-01046-7

**Published:** 2021-02-08

**Authors:** Masako Tada, Ayaka Hayashi, Yumi Asano, Musashi Kubiura-Ichimaru, Takamasa Ito, Miho Yoshii, Hiroshi Kimura, Yoichi Matsuda, Mitsuo Oshimura

**Affiliations:** 1 Stem Cells and Reprogramming Laboratory, Department of Biology, Faculty of Science, Toho University, Miyama 2-2-1, Funabashi, Chiba 274-8510 Japan; 2 Chromosome Engineering Research Center, Tottori University, Yonago, 683-8503 Japan; 3 Cell Biology Unit, Institute of Innovative Research, Tokyo Institute of Technology, Tokyo, 226-8501 Japan; 4 Department of Animal Sciences, Graduate School of Bioagricultural Sciences, Nagoya University, Nagoya, 464-8601 Japan

**Keywords:** DNA methylation, Chicken, Mammalian chromosome, Divergence

## Abstract

**Background:**

DNA methylation is a significant epigenetic modification that is evolutionarily conserved in various species and often serves as a repressive mark for transcription. DNA methylation levels and patterns are regulated by a balance of opposing enzyme functions, DNA methyltransferases, DNMT1/3A/3B and methylcytosine dioxygenases, TET1/2/3. In mice, the TET enzyme converts DNA cytosine methylation (5mC) to 5-hydroxymethylcytosine (5hmC) at the beginning of fertilisation and gastrulation and initiates a global loss of 5mC, while the 5mC level is increased on the onset of cell differentiation during early embryonic development.

**Objective:**

Global loss and gain of DNA methylation may be differently regulated in diverged species.

**Methods:**

Chicken B-cell lymphoma DT40 cells were used as an avian model to compare differences in the overall regulation of DNA modification with mammals.

**Results:**

We found that DNA methylation is maintained at high levels in DT40 cells through compact chromatin formation, which inhibits TET-mediated demethylation. Human and mouse chromosomes introduced into DT40 cells by cell fusion lost the majority of 5mC, except for human subtelomeric repeats.

**Conclusion:**

Our attempt to elucidate the differences in the epigenetic regulatory mechanisms between birds and mammals explored the evidence that they share a common chromatin-based regulation of TET–DNA access, while chicken DNMT1 is involved in different target sequence recognition systems, suggesting that factors inducing DNMT–DNA association have already diverged.

## Introduction

The chicken is the most commonly domesticated bird, classified as *Gallus gallus domesticus*. Since the embryos develop in the egg rather than in the uterus, it has been used for a long time in vertebrate development studies as an excellent model organism (Fumihito et al. 1994; Burt and Pourquie 2003). The superclass Tetrapoda is constituted by four-limbed animals, including mammals, birds, reptiles, and amphibians, which diverged from teleosts by the late Devonian, 355 million years ago (Clack et al. 2017). Although birds are genetically distant from mammals, they both belong to the same amniotes and share similar developmental processes. Since the whole genome sequence of chickens has become available in recent years, chickens can be applied to various genomic and epigenetic analyses (Hillier et al. 2004). DNA methylation is a widely conserved epigenetic information used in many species from plants to animals and is essential for cell differentiation and normal embryonic development in mammals (Li et al. 1993; Li and Zhang 2014). DNA methylation may be controlled by mechanisms that are primarily conserved among species (Zhong 2016). However, our understanding of the mechanisms regulating access between DNA and DNA-modifying enzymes in animal species except for mice and humans remains incomplete.

DNA methylation is catalysed by DNA methyltransferases (DNMT) at cytosines in the CpG sequence. DNMT1 functions to add a methyl group to cytosine in the complementary DNA strand of methylated cytosine (5mC) during DNA replication, and the de novo type enzymes DNMT3A and DNMT3B newly add a methyl group to unmodified cytosine in the CpG sequence (Li et al. 1992; Okano et al. 1999). Furthermore, 5mC is oxidised by the TET family of enzymes, TET1, TET2, and TET3 especially in early mouse embryogenesis and converted to 5-hydroxymethylcytosine (5hmC) (Tahiliani et al. 2009; Inoue and Zhang 2011). Genome analysis shows that chicken genomes are rich in 5mC (Li et al. 2011), and include orthologs of mouse DNMT1, 3A, 3B, and TET1, 2, and 3 (https://www.ncbi.nlm.nih.gov/grc/chicken). The chicken DNMT enzymes have been analysed (Yokomine et al. 2006; Lyko 2018) and chicken *TET1–3* were isolated, and the expression profiles were briefly examined in embryonic development and adult tissues (Okuzaki et al. 2017). In adult chickens, *TET* genes are mainly expressed in the lungs, spleen, intestines, oviducts, leukocytes, and erythrocytes. Most curiously, *Tet1* expression is limited in adult mice, but *TET1* is the most expressed gene in *TET1–3* in adult chicken tissues and highly expressed in foetal erythrocytes. Therefore, there are few reports on the coregulation between 5mC, 5hmC, and histone modification in chickens to find common or diverse regulatory mechanisms in amniotes.

Histone modifications are significant in gene regulation, heterochromatin formation, and silencing of virus-derived sequences, which are often associated with DNA methylation profiles (Li and Zhang 2014). The H3K9me3-rich regions are accumulated around pericentric heterochromatin and Y chromosome in mice (Lehnertz et al. 2003; Peters et al. 2003; Bulynko et al. 2006). Besides, pericentromeric repeats called satellite 2 and 3 are accumulated on the limited chromosomes 1, 2, 9, 10, and 16 in humans (Tagarro et al. 1994). Although DNA methylation is less accumulated in pericentric heterochromatin in humans compared to mice, DNA methylation plays an essential role in the genomic stability of human somatic cells (Tuck-Muller et al. 2000; Weisenberger et al. 2005).

In our previous study, we have shown that TET enzymes preferentially convert 5mC to 5hmC in euchromatic regions in mouse embryonic stem cells (ESCs) using immunofluorescence staining (IF) analysis (Kubiura et al. 2012). The evidence suggests that 5mC accumulation in the heterochromatic region is maintained not only by a predominant mechanism but also by a passive mechanism based on the restriction of TET enzyme access to histone H3K9me3-rich heterochromatin. Therefore, IF analyses using the metaphase chromosome provide global and important information on the relation between chromatin and DNA modification. It was of great interest to determine, if the DNA and DNA-modifying enzyme association may control DNA methylation profiles not only in mice but also in chickens.

Chicken chromosomes are classified into two groups: macrochromosomes (MACs) and microchromosomes (MICs) (Ladjali-Mohammedi et al. 1999). It has been reported that MICs are rich in genes and GC contents, while MACs contain few genes, replicate during the latter half of S phase, and localise at the periphery of the nucleus (Schmid et al. 1989; Maruyama et al. 2012). Thus, it was thought that MICs and MACs are euchromatic and heterochromatic, respectively. Besides, the pericentromeric regions of MICs, the terminal region of the long arm of the Z chromosome (Zq ter), and almost all part of the W chromosome contain highly repetitive and heterochromatic repeats. In this study, we tried to determine whether DNA modification is under the regulation of chromatin structure in chickens using the chicken B cell lymphoma cell line DT40 as a model (Baba et al. 1985).

Here, we show that the DT40 cell genome is highly methylated, even though TET1–3 are active. The high level of DNA methylation is positively maintained by inhibiting access of TET enzymes, which may be controlled by the repressive histone modification profiles. Furthermore, human and mouse chromosomes transferred into DT40 cells are not targeted by chicken DNMT enzymes and become hypomethylated, except for the subtelomeric regions of the introduced human chromosome. Subtelomeres located at the proximity regions inside the arrays of telomeric repeats are less evolutionary conserved than telomeres, and highly methylated by DNMT to prevent inter- and intra-chromosomal recombination (Gonzalo et al. 2006; Calderón et al. 2014). Our work illustrates that chickens and mammals share the same chromatin-based regulation for DNMT and TET to access to the target DNA, but DNMT1 is involved in evolutionarily divergent target sequence recognition mechanisms.

## Methods

### Cell lines and cultures

We used chicken B cell lymphoma cell line DT40. In addition, we used two DT40 cell lines containing one human chromosome 4 (DT40 + hCh4) or one mouse chromosome 11 (DT40 + mCh11), which were created via microcell-mediated cell fusion (Oshimura et al. 2015). Chicken DT40 cells were cultured in RPMI medium 1640 (Gibco^®^, Thermo Fisher Scientific, Waltham, MA, USA), 10% foetal bovine serum (FBS, Corning, NY, USA), 0.1% chicken serum, 0.1 mM β-mercaptoethanol (2ME, Sigma-Aldrich, St. Louis, MO, USA). Cells were grown under 5% CO_2_ at 39˚C. Mouse J1 ESCs were cultured on the feeder cells in DMEM F12 (Wako, Osaka, Japan) supplemented with 10% FBS, 0.1 mM nonessential amino acid and sodium pyruvate (Invitrogen, Thermo Fisher Scientific, Carlsbad, CA, USA), penicillin–streptomycin and 2 mM l-glutamine (Gibco^®^), 0.1 mM 2ME, and 10^3^ U/ml ESGRO^®^ LIF (Merck, Kenilworth, NJ, USA). Feeder cells were mitomycin C-treated mouse embryonic fibroblasts (MEF) derived from embryonic day 12.5. Human female iPSCs (KAC, Kyoto, Japan) were cultured on feeder cells in DMEM F12 HAM (Wako) supplemented with 20% KnockOut Serum Replacement (KSR™, Thermo Fisher Scientific), 0.1 mM nonessential amino acid, 2 mM L-glutamine, 1 × penicillin–streptomycin, 0.1 mM 2ME, and 5 ng/ml recombinant human basic FGF (Wako). Mouse and human cells were grown under 5% CO_2_ at 37 °C.

### Inhibitor treatment

The HDAC inhibitors trichostatin A (TSA, Sigma-Aldrich) and valproic acid (VPA, Sigma-Aldrich) were dissolved in dimethylsulfoxide (DMSO, Sigma-Aldrich) and PBS, respectively. The stock solution was filter sterilised and stored at − 20 °C. Cells were cultured in the medium containing 0.3 µM TSA or 2 mM VPA at 39 °C for six hours (h).

### Chromosome preparation

Mitotic cells were enriched by treatment with 0.3 μg/ml colcemide (Demecolcine, Sigma-Aldrich) for 1 h before harvesting. DT40 cells and human iPSCs were treated with 0.075 M KCl for 20 min, while mouse ESCs were treated for 7 min at room temperature, followed by the addition of 1/10 volume of a 3:1 mixture of methanol and acetic acid. Cells were then fixed three times, with 100% of the methanol and acetic acid fixative. Chromosome spreads were prepared on glass slides using a standard air-drying method. Chromosomal double-stranded DNA was denatured in 4 N HCl for 8 min at room temperature.

### IF analysis for 5mC and 5hmC

Careful attention was paid to DNA denaturation efficiency to rule out the possibility that the 5hmC-negative chromosomal region was inaccessible to the anti-5hmC antibody due to the technical insufficiency. It is worth noting that methanol: acetic acid mixture was used instead of the paraformaldehyde fixative to remove chromosomal proteins that inhibit efficient DNA denaturation. The fact that most of the 5hmC-negative regions, especially around the centromere, is conversely positive for 5mC shows that the IF method is sufficient to detect 5mC and 5hmC without technical problems. Also, the chromosomal preparation and DNA denaturation used in this study are sufficient for DNA probes to efficiently hybridise to satellite repeats around the centromere, which was shown using fluorescence in situ hybridisation (Kubiura et al. 2012). The denatured chromosome samples were permeabilised with PBS-Triton X for 10 min at room temperature, and pre-blocked in 2% skim milk/PBS (Gibco^®^, Thermo Fisher Scientific, Waltham, MA, USA) for 30 min at room temperature. It was then incubated in mouse monoclonal anti-5mC antibody (1:500, Active Motif) and rabbit polyclonal anti-5hmC (1:500, Active Motif, Carlsbad, CA, USA) diluted in 2% skim milk/PBS for 1 h at room temperature. The IF signal was detected with anti-mouse IgG H&L Alexa Fluor^®^ 488 (1:500) and anti-rabbit IgG H&L Alexa Fluor^®^ 546 (1:500) (Invitrogen, Carlsbad, CA, USA), respectively. After the antibody treatment, the sample was washed three times, with 0.05% Tween 20/PBS (PBST). DNA was then stained with Prolong Gold antifade reagent containing DAPI (Thermo Fisher Scientific). Images of nuclei and chromosomes were obtained using an LSM780 confocal microscope (Carl Zeiss, Oberkochen, Germany).

The mean and standard deviation of the fluorescence intensity per area was calculated using total fluorescence intensity for 5mC, 5hmC, and DAPI in each chromosome spread. Each value of total fluorescence intensity was calculated using Image J software, version: 2.0.0-rc-69/1.52p (an open-source program for image analysis provided by the National Institutes of Health, Bethesda) (Schindelin et al. 2012). The triple knockout mouse ESCs for *Dnmt1*, *Dnmt3a*, and *Dnmt3b* (DNMT TKO ESCs) were used as a negative control for the 5mC and 5hmC IF staining, of which values obtained were set to 0.

### IF analysis for histone H3K9me3 and H3K4me3

After hypotonic treatment, cells were applied to each poly-l-lysine-coated 12-mm round coverslip (BD Biosciences) that had been placed on the bottom of the cylindrical tube. Then, the tube was centrifuged for 3 min at 800 rpm. Chromosome spreads and nuclei attached to the coverslips were directly treated with 2% skim milk/PBS at room temperature for 30 min. Samples were incubated with primary antibody overnight at 4 °C. The anti-mouse monoclonal antibody for H3K9me3 (Hayashi-Takanaka et al. 2011) and the anti-rabbit polyclonal antibody for H3K4me3 (1:1000, Abcam, Cambridge, UK) were applied to native chromosomes. After being washed three times with PBST, the coverslips were fixed with 2% PFA/PBS for 5 min. The IF signal was detected as described above.

### Slot blotting of genomic DNA

Genomic DNA samples were isolated from cultured cells and chicken embryonic tissues of the WL-G strain according to a widely used procedure. Fifteen micrograms of DNA from each sample were dissolved in Tris–EDTA (TE) buffer and fragmented to around 500 bp using Bioruptor (Cosmo Bio, Tokyo, Japan), and then 500 ng of fragmented DNA was denatured at 100 °C for 10 min for each sample. After immediate cooling on ice, denatured DNA samples were transferred onto Hybond^R^-N^+^ nylon membranes (GE Healthcare, Barrington, IL, USA) using Bio-Dot Microfiltration Apparatus (Bio-Rad Laboratories, Hercules, CA, USA). Membranes were presoaked in 6 $\times$ SSC for at least 10 min, and then TE buffer was applied to all sample slots and allowed to flow through. The denatured DNA sample was then aspirated and attached to the membrane. After rinsing the blotted membrane with 2 $\times$ SSC, the membrane was baked at 80 °C for 2 h.

### Quantification of 5mC and 5hmC

The blotted membranes were pre-treated with 3% skim milk/PBST for 30 min, and then incubated overnight at 4 °C in mouse monoclonal anti-5mC antibody (1:5000) or rabbit polyclonal anti-5hmC (1:10,000) diluted in 3% skim milk/PBS. Hybridised bands were detected by secondary antibody treatment with HRP-mouse IgG (1:3000) or HRP-rabbit IgG (1:5000), respectively, and visualised using the ECL Prime Western blotting detection kit (GE Healthcare). The relative densities of the signals were analysed using an ImageQuant™ LAS-4000 mini imaging system (GE Healthcare).

### Fluorescence in situ hybridisation (FISH)

Mouse Cot1 DNA and human Cot1 DNA (Invitrogen™, Thermo Fisher Scientific) were labelled with Digoxigenin-11-dUTP (Roche, Basel, Switzerland) using a standard nick translation kit (Roche). DNA FISH was performed according to the procedure previously described (Kubiura et al. 2012). Hybridised signals were detected using anti-DIG-Rhodamine (1:100). Chromosomal DNA was counterstained with DAPI (Thermo Fisher Scientific). FISH signals were detected using an Axio Imager-Z2 microscope (Carl Zeiss).

### RT-qPCR analysis

Total RNA was extracted using the RNeasy Plus Mini Kit (Qiagen, Hilden, Germany). First-strand cDNA was synthesised from 0.5 µg of RNA using SuperScript III reverse transcriptase (RT, Thermo Fisher Scientific), and cDNA derived from 10 ng total RNA was used for one reaction of quantitative polymerase chain reaction (qPCR). RT-qPCR was performed using Power SYBR (Thermo Fisher Scientific) on the LightCycler 480 system (Roche). ACTB, a housekeeping gene, was used as an internal control. Primers used were listed in Table 1.

**Table 1:** PCR primers used for gene expression analyses

Set	Gene	Primer name	Sequence 5′–3′	Size	
1	*DNMT1*	qRTchDNMT1-F	acggcttcttcagcaccacggtc	168	NM_206952.1
		chDNMT1-R	ctgtcggtgtttatccaggatgtt		
2	*DNMT3A*	qRTchDNMT3A-F	ggtacttctggggtaaccttcc	194	NM_001024832.1
		chDNMT3A-R	tccttctcgttcatgaagacgg		
3	*DNMT3B*	chDNMT3B-F	tcctactgcaccgtgtgctg	150	NM_001024828.3
		chDNMT3B-R3	tagcagttccagggctcctgc		
4	*TET1*	qRTchTET1-F	aaagatgaaggcccgtattac	149	XM_025151680.1
		qRTchTET1-R	gagagctttttccttcctttc		
5	*TET2*	qRTchTET2-F	tgtgcttgtcaagggctggaccc	104	NM_001277794.1
		qRTchTET2-R	tcttgcttctggcaaacttaca		
6	*TET3*	qRTchTET3-F	ccgcatcgagaaggtcatctac	162	XM_015297468.2
		qRTchTET3-R	ggatgatgatgacagcattctg		
7	*ACTB*	qRTchBACTIN-F	attggcaatgagaggttc	77	NM_205518.1
		qRTchBACTIN-R	ggataccacaggactccat		

### Statistics

Data are presented as mean ± standard deviation. The *Z* test Excel formula was used to evaluate the statistical significance of the differences between two samples. Significance was determined using equal-variance *Z* values on both sides. Values of *p* < 0.01 were considered significant. *P < 0.05; **P < 0.01; ***P < 0.001.

### Ethical statement and approval for animal experiments

The authors declare that chicken fertilised eggs were used under the research policy of Toho University.

## Results

### Global localisation of DNA modification over chromosomes in chicken DT40, human iPSCs, and mouse ESCs

**Fig. 1:**
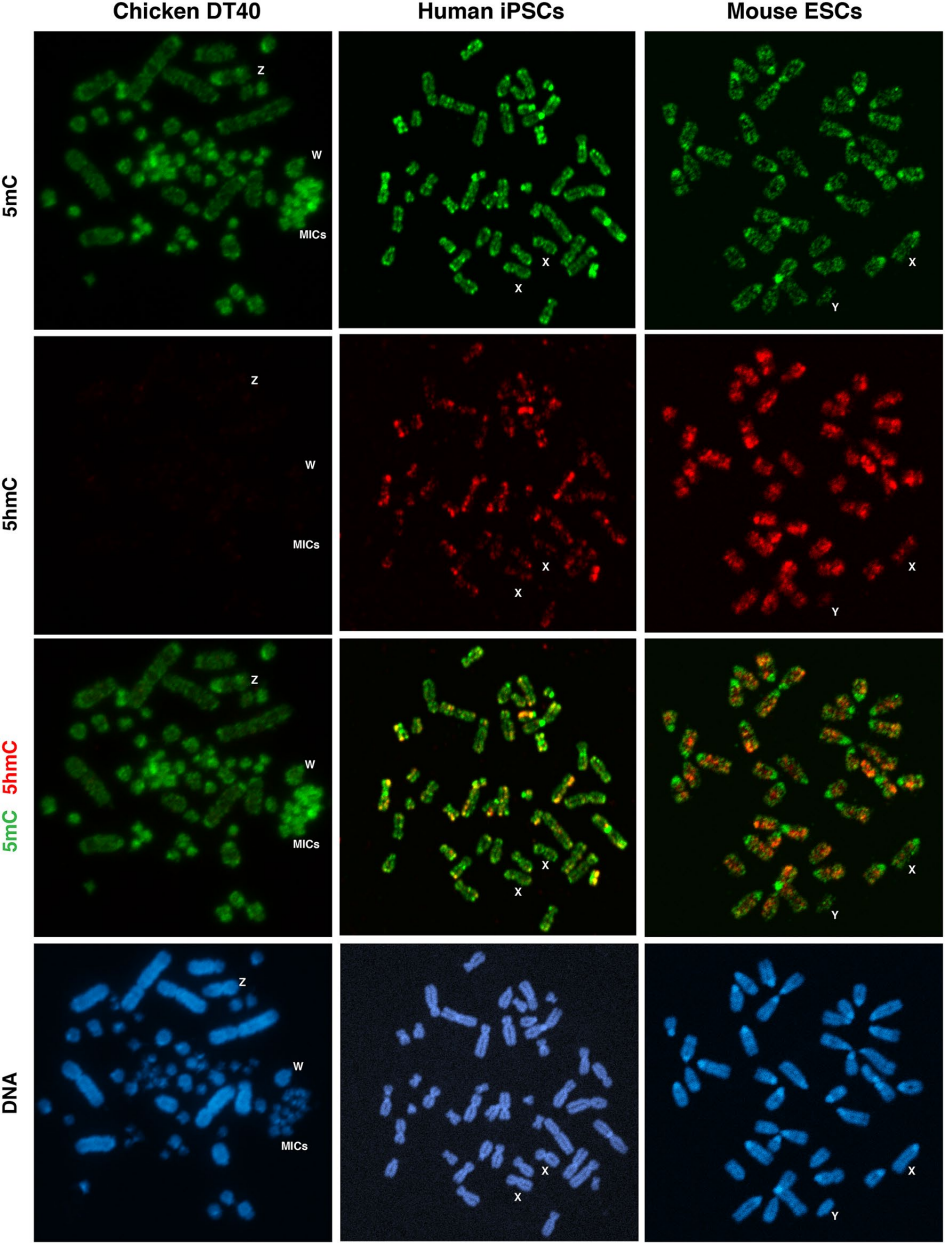
Global localisation of DNA modification over chromosomes in chicken DT40 cells, human iPSCs, and mouse ESCs. Two-colour immunofluorescent (IF) analysis of 5-methylcytosine (5mC, green) and 5-hydroxymethylcytosine (5hmC, red). DNA stained with DAPI (blue) shows a G-band pattern on chromosomes (colour figure online)

First, we compared 5mC and 5hmC distribution patterns across metaphase chromosomes in chicken, humans, and mice using DT40 cells (80, ZW), human induced pluripotent stem cells (iPSCs, 46, XX), and mouse ESCs (40, XY) by means of the IF method. It is worth noting that human iPSCs are classified into the primed state of pluripotency and are at a more advanced stage than mouse ESCs. Therefore, the TET enzyme is less expressed in human iPSCs, resulting in being less positive for 5hmC in cells, compared with mouse ESCs. On the other hand, the repeat sequences on the MIC, W, and Z of chicken chromosomes are strongly positive for 5mC, while all chicken chromosomes are negative for 5hmC (Fig. 1). There were two possible reasons for this to occur in DT40 cells: repression of *TET* expression or access restriction of TET enzymes to the methylated CpG sequences.

### Limited DNA demethylation via TET enzymes in chicken DT40 cells

**Fig. 2:**
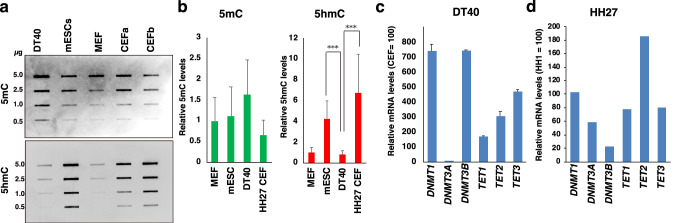
Limited DNA demethylation via TET enzymes in chicken DT40 cells. **a** A representative slot-blot image for 5mC and 5hmC, using genomic DNA of five samples: chicken DT40 cells, mouse ESCs (mESCs), mouse embryonic fibroblasts (MEF), chicken embryonic fibroblasts from two HH27 embryos (CEFa and b). **b** Histograms for 5mC and 5hmC show the relative signal intensities among the samples (MEF = 1, n = 4). **c** RT-qPCR analysis of the gene expression of the *DNMT* and *TET* family genes in DT40 cells and HH27 CEF (n = 2, triplicated measures). Each value was normalised to the average value of HH27 CEFs, set as 100. **d** GEO DataSet Analysis (GSE28388) was performed for the *DNMT* and *TET* family gene expression in HH1 and HH27 embryos (n = 2). Each value was normalised to the average value of HH1, set as 100

To define whether the TET family of enzymes generally functions in chicken cells as seen in mice, we performed slot dot blot analysis for 5hmC using genomic DNA from chicken and mouse cells (Fig. 2a). The luminescence signal intensity was measured, and the relative intensity was assessed by setting the mouse embryonic fibroblast (MEF) signal intensity as 1 (Fig. 2b). Genomic DNA obtained from DT40 cells was more methylated than DNA from mouse ESCs, MEFs, and chicken embryonic fibroblasts (CEFa and b, *n* = 2) obtained from two 6-day-old embryos at Hamburger and Hamilton (HH) stage HH27. In addition, 5hmC was detected in HH27 chicken fibroblasts but detected in DT40 cells at a very low level (Fig. 2a, b). Because early mouse embryonic cells are rich in TET activity (Hackett et al. 2013; Ruzov et al. 2011), similar tissue-specific gene expression regulation and target recognition mechanisms may be shared by chickens and mammals.

The chicken genome has a size of 1.05 Gbps, while the mouse genome has a capacity of 2.5 Gbps, with 15% and 45% of each being repetitive sequences, respectively (Faulk et al. 2016; Wicker et al. 2005). If repeat sequences are fully methylated in both animal groups, mouse genomes should be methylated threefold more than chicken genomes. However, the result from DNA slot-blot hybridisation for 5mC demonstrates that chicken and mouse somatic genomes are methylated at a similar extent (Fig. 2a, b, *n* = 4, *p* = 0.293). On the contrary, the result from DNA slot-blot hybridisation for 5hmC demonstrates that DT40 genomes are significantly less hydroxymethylated than mouse ESCs and HH27 chicken embryonic fibroblasts (*n* = 2) (Fig. 2a, b, *p* < 0.001). Therefore, mouse ESC genomes may be abundantly methylated more than chicken DT40 cells, but some of this 5mC is converted to 5hmC and unmethylated cytosine. In chickens, TET family enzymes can access DNA under a developmental regulation and demethylate HH27 genomes.

Next, we examined the expression of genes for DNA modifying enzymes using RT-qPCR analysis. Our results demonstrate that *DNMT1*, *DNMT3B*, and *TET1-3* are abundantly expressed in DT40 cells, more so than in HH27 CEFs (Fig. 2c). The microarray data set published in the GEO DataSet Analysis (GSE28388) (Irie and Kuratani 2011) shows that *DNMT1* is rather consistently expressed throughout chicken embryonic development from HH1 to HH27 (Fig. 2d). Therefore, DNMT1 and DNMT3B are possibly major DNMTs working in DT40 cells. Furthermore, the low level of 5hmC IF signals in DT40 cells is not due to transcriptional repression of *TET1–3*, all of which are highly expressed in those cells, more than in fibroblasts from early chicken embryos (Fig. 2c, d). It is noteworthy that *TET1* was not the most highly expressed gene in *TET1–3* in DT40 cells and embryos.

### Limited DNA demethylation via TET enzymes by compact chromatin in chicken DT40 cells

**Fig. 3:**
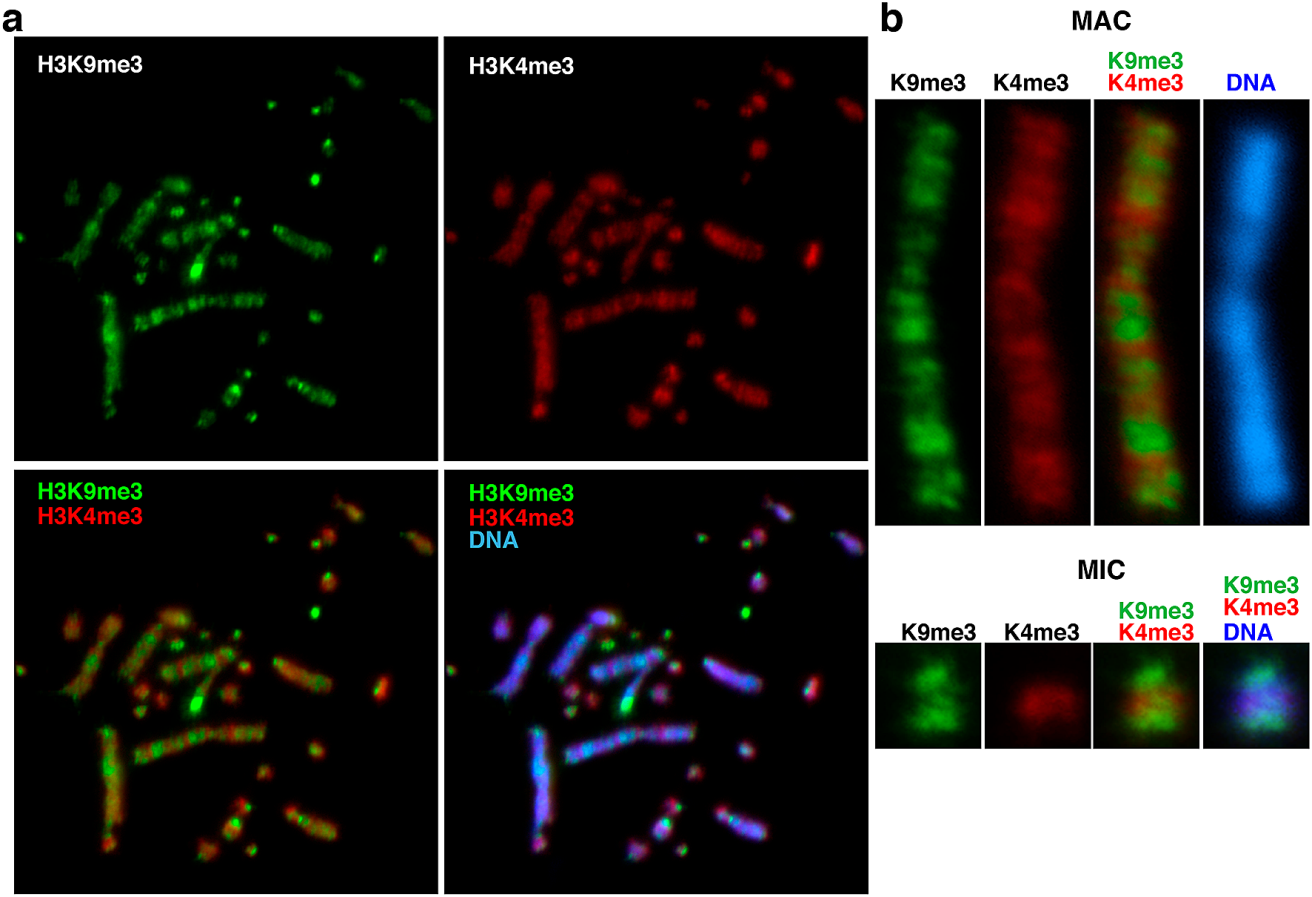
Reciprocal localisation of H3K4me3 and H3K9me3 across chicken chromosomes in DT40 cells. **a** Two-colour IF analysis of histone H3 trimethylated at lysine 9 (H3K9me3, green) and H3K4me3 (red), which are representative epigeneic marks for heterochromatin and euchromatin, respectively. **b** Enlarged images of a macrochromosome (MAC) and a microchromosome (MIC). Chromosome bands stained with DAPI (blue) are mostly positive for H3K9me3 (green) (colour figure online)

TET-DNA association could be regulated mainly by chromatin structure, not by a difference in levels of DNA-modifying enzymes expressed in DT40 cells. Before addressing this possibility, we investigated the distribution of H3K4me3 and H3K9me3 over DT40 chromosomes. These are the most well-known representative histone marks located in transcriptionally active and inactive regions, respectively. Both of them are present throughout the DT40 chromosome. The reciprocal relation of H3K9me3 and H3K4me3 produces a banding pattern on MACs and MICs (Fig. 3). In addition, we found that even in the H3K4me3-positive region, genomes were entirely negative for 5hmC in DT40 cells (Figs. 1, 3). The H3K4me3-positive regions usually escape from the DNMT access (Neri et al. 2013). Exclusion of DNMT from the active promoter regions may be the reason why 5hmC, an oxidation form of 5mC, may not be detected even if TET is accessible to this region.

**Fig. 4:**
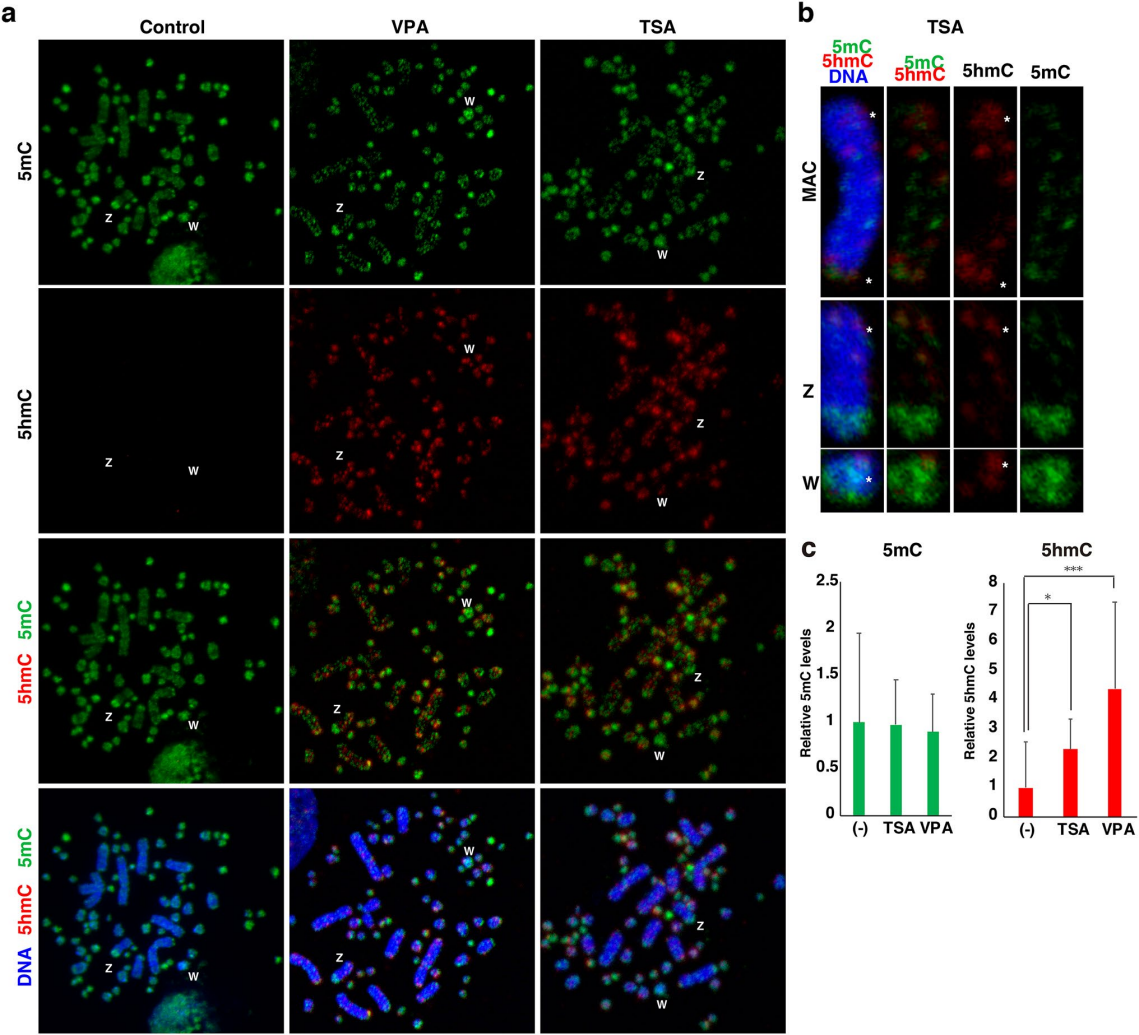
Restriction of TET-mediated DNA demethylation by repressive chromatin in chicken DT40 cells. **a** DT40 cells were cultured for six hours in a medium containing 2 mM trichostatin A (TSA) or 0.3 nM valproic acid (VPA). 5hmC signal (red) became detectable in DT40 chromosomes. **b** Enlarged images of a macrochromosome (MAC), Z and W chromosomes. Highly condensed heterochromatin located in the half of Zq ter and the most part of W were still protected from hydroxymethylation. In contrast, subtelomeric regions, located close to the chromosome end, were positive for 5mC and 5hmC (asterisks). **c** Global increase in 5hmC levels in cells cultured for six hours in a medium containing TSA or VPA (colour figure online)

In mouse ESCs, TET access is excluded from H3K9me2/3-marked regions (Kubiura et al. 2012). Therefore, in order to verify that TET can access the 5mC regions when chromatin is opened, DT40 cells were treated with trichostatin A (TSA) and valproic acid (VPA). These compounds are widely used as the histone deacetylase (HDAC) inhibitors, and are known to activate chromatin by increasing histone acetylation levels (Hezroni et al. 2011). As a result, 5hmC was detected in DT40 cells treated with TSA and VPA for six hours under the comparative conditions to the cells shown in Fig. 1 (Fig. 4a), especially around subtelomeric repeats (Fig. 4b, asterisks). However, the heterochromatic regions of the Zq ter and W were still negative for 5hmC (Fig. 4b). The 5hmC IF signal levels were increased 2- and fourfold over untreated DT40 cells by culturing cells in a medium containing TSA and VPA, respectively (Figs. 1, 4c). Therefore, our results suggest that DT40 cells possess sufficient TET enzyme activity, but the repressive chromatin maintains a high level of 5mC status by continuous restriction of the TET access.

### The target-sequence recognition property of chicken DNMT enzymes in DT40 cells

**Fig. 5:**
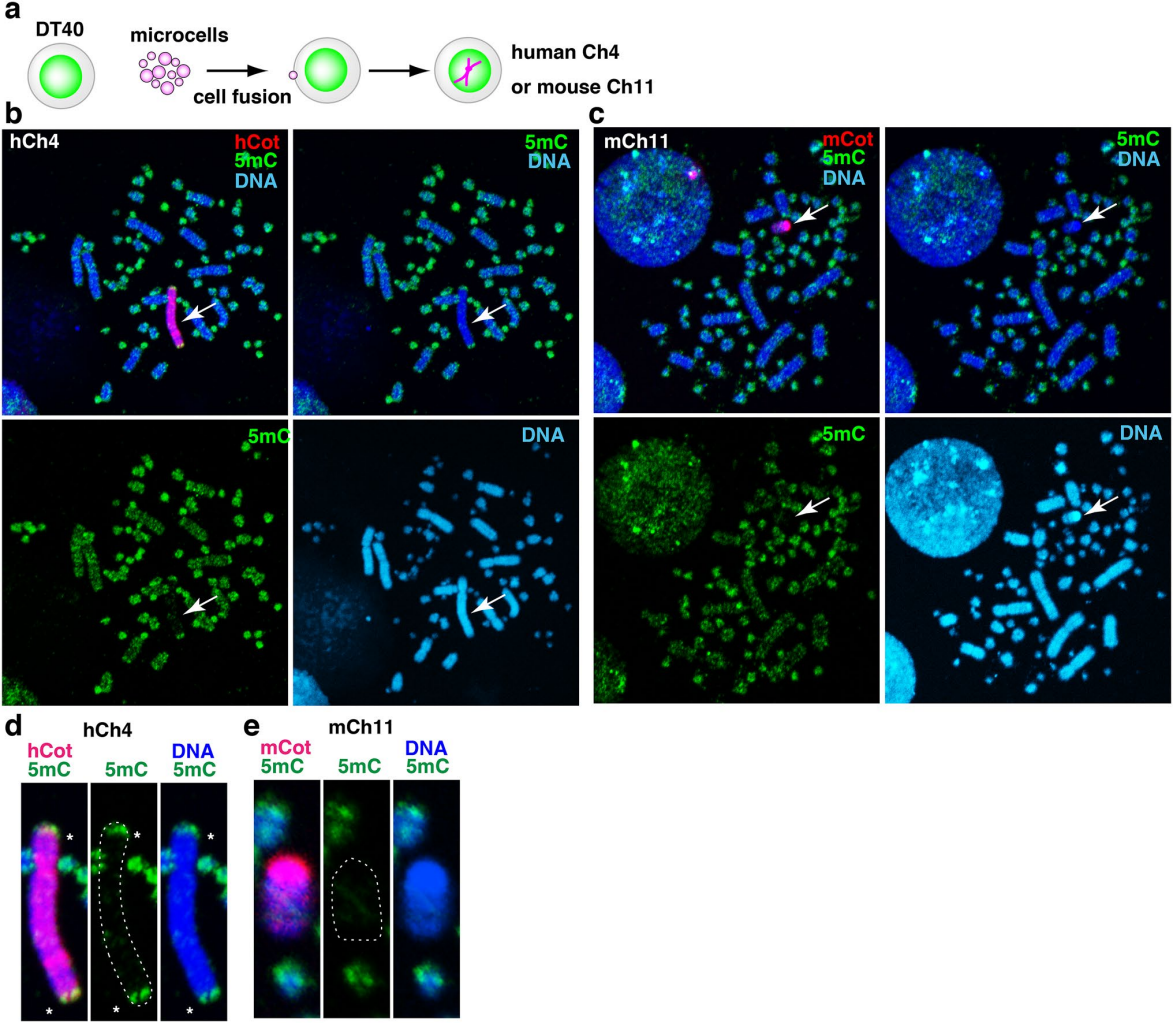
Target sequence recognition by chicken DNMT enzymes in DT40 cells containing one human or mouse chromosome. **a** An experimental scheme for the production of hybrid DT40 cells either carrying a human chromosome 4 (hCh4 DT40) or a mouse chromosome 11 (mCh11 DT40). **b** Two-color detection of in situ fluorescent hybridisation (FISH) signals for human Cot1 repeats (hCot1, red) and IF signals for 5mC (green) in hCh4 DT40 cells. **c** FISH signals for mouse Cot1 repeats (mCot1, red) and IF signals for 5mC (green) in mCh11 DT40 cells. DNA was stained with DAPI (blue). **d** Enlarged images of hCh4. **e** Enlarged images of mCh11 (colour figure online)

Finally, to address the question of how the two diverged animals share the mechanisms underlying the regulation of DNA methylation profiles, we analysed interspecific hybrid cells between chicken and humans or mice. To investigate whether chicken DNMT and TET could maintain the previous DNA modifications located on mammalian genomes, we used two hybrid DT40 cell lines that contain one human chromosome 4 or one mouse chromosome 11 (Fig. 5a). These cells are abbreviated, hCh4 DT40 cells and mCh11 DT40 cells, respectively. The two trans-chromosomic hybrid cell lines have been created via microcell fusion using mammalian somatic cells as a chromosome donner (Oshimura et al. 2015). The DNA methylation status of human and mouse chromosomes was analysed by IF analysis for 5mC and 5hmC in DT40 hybrid cells. In addition, metaphase chromosome preparations were hybridised with digoxigenin-labelled human cot (hCot) and mouse cot (mCot) probes to identify exogenous human and mouse chromosomes in hCh4 and mCh11 DT40 cells, respectively. The FISH signals were co-detected with 5mC IF signals. As a result, most of the introduced human and mouse chromosomes were demethylated in hCh4 and mCh11 DT40 cells (Fig. 5b, c), even though 70% CpG sequences are usually methylated in mammalian somatic cells (Li and Zhang 2014). In contrast, the subtelomeric regions at both ends of human chromosome 4 were highly methylated in a region-specific manner (Fig. 5d). The result suggests that chicken DNMT family enzymes work in trans on exogenous human sequences in DT40 cells to some extent, but they possess target sequence preference. Unlike telomere, the subtelomere sequences vary among species and are methylated in mammalian cells by DNMT, especially DNMT1 and DNMT3B to stem an excess inter-chromatid exchange (Gonzalo et al. 2006; Toubiana and Selig 2020). Therefore, it appears that the chicken DNMT complex can recognize human subtelomere sequences but not the mouse one. Moreover, the chicken DNA methylation system was unable to maintain mouse centromeric DNA methylation in DT40 cells (Fig. 5e). Even assuming that chicken TET enzymes preferentially demethylate mammalian chromosomes introduced into DT40 cells, the unmethylated state of mammalian chromosomes in hybrid cells may be maintained by the failure of re-methylation of mammalian chromosomes by chicken DNMT3B and failure to maintain DNA methylation by chicken DNMT1. Chicken DNMT family enzymes, mainly DNMT1, may have acquired their target sequence preferences, which is often indirectly regulated by a particular binding protein. It is possible that cofactors that bind to DNMT1 may differ in each species to produce DNMT1-DNA proximity at sequence-specific fashion. Therefore, DNA methylation maintenance mechanism is likely to be coevolving together with the DNA sequence divergence.

## Discussion

In this study, we introduced new approaches to the field of epigenetics to find out the general mechanisms involved in the regulation of global DNA modification during embryonic development in animals. Biologically significant systems must be conserved over taxons or replaced by an alternative redundant mechanism. To resolve either conserved or specialised mechanisms to regulate DNA methylation in chickens and mammals, we analysed DNA methylation patterns over metaphase chromosomes in chicken DT40 cells using IF methods. Chromatin activation induced by cell culture in medium supplemented with HDAC inhibitors can convert highly methylated DNA back into 5hmC-containing DNA to some extent. Moreover, when one of the mouse or human chromosomes was introduced, DT40 cells could not maintain most DNA methylation of mouse and human chromosomes, including mouse pericentric heterochromatin, except for human subtelomeric repeat sequences. These results demonstrate that TET target regions are similarly regulated between chickens and mammals, but DNA methylation maintenance mechanism is different between chickens and mammals.

DNMT1 is an abundant protein and interacts with many other proteins, which regulate sequence-specific proximity between DNMT1–DNA during S phase of the cell cycle and maintain DNA methylation profiles mostly in heterochromatic regions (Sharif et al. 2016; Hervouet et al. 2018). Furthermore, de novo DNA methyltransferases DNMT3A and DNMT3B possess active recruitment mechanisms into the specific target regions via associations with other proteins or RNA (Li and Zhang 2014; Cedar and Bergman 2009; Laisné et al. 2018). Therefore, the divergence of not only DNMT1 itself but also many other DNMT1-binding proteins possibly contributes to the species-specific regulation in DNA methylation. In addition, the results may suggest an evolutional aspect of the process of establishing new repetitive sequences. Young repeats first amplify the sequence by tandem duplication or translocation, most of which might later have been inactivated by a coevolved mechanism. Target-sequence regulation by DNMT may play a key role to serve a protection mechanism of the genome from the further detrimental accumulations during germline development. Accordingly, we propose the possibility that the DNMT-associating factor(s) that recognise sequence-specific target sequences may already far diverge in chickens and mammals.

As mentioned earlier, chicken embryos develop in the egg rather than in the uterus, which is the reason why chicken is a suitable model system for various studies in the embryonic development process. In the laid eggs, embryonic development is arrested at the blastoderm stage. Primitive streak formation can be triggered in a fertilised egg by its incubation at 39–40 °C, which in turns leads to mesendoderm formation. This process is a key developmental process that diverges blastoderm into three germ layers and germ cells (Hamburger and Hamilton 1992). Therefore, our IF-based epigenetic analysis applied to metaphase chromosomes will provide information to resolve how chromatin structure and DNA methylation are co-regulated in vivo every hour and every minute during the epigenetic reprogramming process in chickens. We are currently working on this issue.

## Data Availability

All data discussed in this publication is included in this manuscript. Further information and requests for data and reagents should be requested to the corresponding author, Masako Tada. Please contact masako.tada@sci.toho-u.ac.jp.
